# Atypical Resting-State Functional Connectivity Dynamics Correlate With Early Cognitive Dysfunction in HIV Infection

**DOI:** 10.3389/fneur.2020.606592

**Published:** 2021-01-14

**Authors:** Benedictor Alexander Nguchu, Jing Zhao, Yanming Wang, Yu Li, Yarui Wei, Jean de Dieu Uwisengeyimana, Xiaoxiao Wang, Bensheng Qiu, Hongjun Li

**Affiliations:** ^1^Hefei National Laboratory for Physical Sciences at the Microscale, Centers for Biomedical Engineering, University of Science and Technology of China, Hefei, China; ^2^Department of Radiology, Beijing Youan Hospital, Capital Medical University, Beijing, China; ^3^School of Biological Science and Medical Engineering, Beihang University, Beijing, China

**Keywords:** fMRI, temporal variability, HIV-associated neurocognitive disorders, brain resting-state functional connectivity dynamics, functional connectivity

## Abstract

**Purpose:** Previous studies have shown that HIV affects striato-cortical regions, leading to persisting cognitive impairment in 30–70% of the infected individuals despite combination antiretroviral therapy. This study aimed to investigate brain functional dynamics whose deficits might link to early cognitive decline or immunologic deterioration.

**Methods:** We applied sliding windows and K-means clustering to fMRI data (HIV patients with asymptomatic neurocognitive impairment and controls) to construct dynamic resting-state functional connectivity (RSFC) maps and identify states of their reoccurrences. The average and variability of dynamic RSFC, and the dwelling time and state transitioning of each state were evaluated.

**Results:** HIV patients demonstrated greater variability in RSFC between the left pallidum and regions of right pre-central and post-central gyri, and between the right supramarginal gyrus and regions of the right putamen and left pallidum. Greater variability was also found in the frontal RSFC of pars orbitalis of the left inferior frontal gyrus and right superior frontal gyrus (medial). While deficits in learning and memory recall of HIV patients related to greater striato-sensorimotor variability, deficits in attention and working memory were associated with greater frontal variability. Greater striato-parietal variability presented a strong link with immunologic function (CD4+/CD8+ ratio). Furthermore, HIV-infected patients exhibited longer time and reduced transitioning in states typified by weaker connectivity in specific networks. CD4+T-cell counts of the HIV-patients were related to reduced state transitioning.

**Conclusion:** Our findings suggest that HIV alters brain functional connectivity dynamics, which may underlie early cognitive impairment. These findings provide novel insights into our understanding of HIV pathology, complementing the existing knowledge.

## Introduction

Despite combination antiretroviral therapy (cART) use, 30–70% of the HIV-infected individuals develop neurocognitive disorders (HAND) (1–3). The brain-communicating systems corresponding to cognitive and behavioral functions are susceptible to pathological features of HIV infection (4, 5). Neurocognitive deficits for HIV patients include attention and working memory, motor control, visuospatial processing, and executive functioning (6). The neural mechanisms underlying these impairments remain largely unclear. It has been shown that magnetic resonance imaging (MRI) can be sensitive to brain changes linked to impairments (1, 7–9).

Gray matter loss in sensorimotor cortices and basal ganglia (striatum) has been reported in patients with HIV (10–12). This volume deficit is overly associated with CD4+ lymphocyte counts and HAND in severely immunosuppressed patients (13, 14). Alterations of striato-cortical regional activities (ReHo) and amplitude of low-frequency fluctuations (ALFF) relating to patients' CD4+T-cell counts and waning cognitive learning and memory have also been reported (15, 16). Studies of asymptomatic HIV patients, however, have reported that antiretroviral drugs could delay or improve brain activity (17). For example, switching from efavirenz to rilpivirine appeared to improve functional connectivity of the dorsal attention network along with enhancing working memory, speed of visual processing, and executive function while switching from raltegravir to dolutegravir appeared to improve the dorsal attention and associative visual and sensory-motor networks (17). Studies have also suggested that assessing functional interactions of multiple regions could reveal more brain changes underlying brain functionality losses in HIV patients (18).

Now, studies focused on resting-state functional connectivity (RSFC) analyses have found attenuation in striato-cortical functional connectivity in HIV patients (19, 20). The most consistent impaired functional connectivities involved striatal, lateral prefrontal, and parietal regions, and regions of the default mode network (DMN) (19, 20). In cortical regions, altered connectivity was reported within PFC areas and between PFC and parietal cortex (19–22). The salience and executive networks also demonstrated intrinsic hypo-connectivity, but hyper-connectivity was found between regions of DMN and regions of frontoparietal and sensorimotor networks (23, 24). These changes were related to deteriorating cognitive functions (22, 23). Emerging research suggests that brain interactions are inherently dynamic and thus studying dynamic properties of these interactions could provide more insight into disease pathology (25–27).

A full-scale characterization of complex dynamic processes may be difficult, but low-scale dynamics such as temporal variability and properties of states of reoccurrence of dynamic RSFC may describe disease pathology beyond static RSFC (28, 29). Deficits in these properties have already been reported in patients with major depression (30), Parkinson's disease (31), autism (32), and schizophrenia (33, 34). For example, patients with Parkinson's disease showed within-network sparse connectivity in states with shorter dwelling time (the duration spent by the participant in a certain state) (31). On the other hand, patients with schizophrenia exhibited a longer time in hypo-connected states but a shorter time in hyper-connected states (33, 35). While temporal variability, the standard deviation of correlation across windowed time, is generally considered to reflect levels of brain adaptability and flexibility relating to learning (32), prefrontal variability is also involved in thinking and planning, a property which is altered in major depression (28, 36). Together with other studies (37, 38), these findings suggest that dynamic RSFC could reliably capture cognitively relevant pathogenic features.

In this study, an attempt is made to detect HIV-associated dynamic changes that may underlie persisting cognitive impairments. With sliding windows and K-means clustering applied to the fMRI data of HIV-infected patients adhering to cART but presenting asymptomatic neurocognitive impairment (ANI) and healthy controls, we construct the whole-brain dynamic RSFC maps and further assess the strength (average) and temporal variability of dynamic RSFC, and state properties: state network topology; mean dwelling time; and state transitions—the number of times a switch occurs from one state to the other. The current study is designed to capture a wide range of state transitions, including specific-state-to-specific-state transitioning in an effort to identify cognitively relevant state transitioning changes (39). We speculate that HIV impairs functional dynamics and that dynamic RSFC deficit (temporal variability or state transitioning) may be related to disease severity (the decline of CD4+/CD8+ ratio and CD4+ T-cell counts) and cognitive performances, suggesting their role in early HAND.

## Materials and Methods

### Subjects

Sixteen HIV-infected patients (mean age ± standard deviation = 30.31 ± 7.21 years; 16 male) with asymptomatic neurocognitive impairment (early HAND) and 16 age- and gender-matched healthy controls (33.00 ± 5.51 years; 16 male) participated in the current study. Neuroimaging and clinical assessment data were collected at the Beijing YouAn Hospital, the Capital Medical University between March 2016 and November 2016. HIV-1 seropositivity with ANI in sustained stable cART regimen [tenofovir (TDF) + lamivudine (3TC) + efavirenz (EFV)] for at least 12 weeks was an inclusion criterion for HIV-participants. Any record of illicit drugs and alcohol use, cerebral atrophy, brain lesions, head injury, or neurological disorders was the other factor for exclusion.

### Blood Test Results and Clinical Neuropsychological Assessments

The clinical assessment characteristics are summarized in Table 1. Given that CD4+ T-cell counts, CD4+/CD8+ ratio, and plasma viral loads were most closely associated with earlier alterations of the brains of HIV patients and are considered to be potential predictors of HIV disease severity (9, 16, 22, 40), we assessed these parameters.

**Table 1:** Demographic and clinical assessments of HIV patients and healthy controls.

**Category**	**HIV patients (*n* = 16)**	**Healthy controls (*n* = 16)**	***P*-value**
Age (years)	30.31 ± 7.21	33.00 ± 5.51	0.246
Gender (male %)	16 (100%)	16 (100%)	
Duration of HIV infection (months) (IQR)	18.5 (12.0–30.0)	ℓ	
CD4+ cell count (cells/μl) (IQR)	466.64 ± 214.44 (104.0–848.0)	ℓ	
CD4^+^/CD8^+^ ratio (IQR)	0.57 ± 0.45 (0.15–1.97)	ℓ	
Undetectable viral load	11 (69%)	ℓ	
Learning and recall (memory) score	36.83 ± 5.87 (28.5–45.5)	ℓ	
Motor score	43.44 ± 8.81 (27–57)	ℓ	
Abstract/executive score	53.42 ± 6.60 (42.5–63)	ℓ	
Verbal and language score	38.44 ± 6.19 (29–57)	ℓ	
Attention/working memory score	37.73 ± 6.31 (24–47)	ℓ	
Information processing speed score	41.19 ± 7.03 (29–52)	ℓ	

*Data are shown in mean ± SD; IQR, interquartile range; ℓ, not measured; and P-values were computed based on two-sample t-test*.

The evaluation of HAND severity was achieved by referring to the Frascati criteria (2). Each patient received a battery of neuropsychological tests for HAND 2 h before the MRI scan. The performances for six cognitive domains were evaluated: (1) learning and recall was assessed using the Hopkins Verbal Learning Test-Revised (HVLT-R) and the Brief Visuospatial Memory Test-Revised (BVMT-R); (2) motor function using the Grooved Pegboard test; (3) abstract and executive function by the Wisconsin Card Sorting Test-64 (WCST-64); (4) information processing speed by the trail making test part A; (5) verbal and language using category fluency and animal naming test; and (6) attention and working memory using the Continuous Performance Test Identical Pairs (CPT-IP), the Wechsler Memory Scale-III (WMS-III), and Paced Auditory Serial Addition Test (PASAT) (also see Supplementary Table 2). Test scores were standardized in T-score. For a cognitive domain tested by multiple testing methods, T-scores were averaged across tests to obtain a composite T-score for the specific domain. A patient was thus considered to have ANI if at least two cognitive domains were impaired (performance of at least 1 SD below the mean for norms on neuropsychological tests) without decreased everyday functioning (2).

### Imaging Procedure and Image Pre-processing

All imaging was conducted on a 3T (Siemens) Scanner (Allegra, Siemens Medical System, Erlangen, Germany). The protocol includes high-resolution T1-weighted anatomical images. fMRI data were collected with an echo-planar imaging (EPI) sequence (see Supplementary Table 1 for imaging parameter settings).

Data were pre-processed using Data Processing and Analysis for Brain Imaging (DPABI V3.0, http://rfmri.org/) (41) and MATLAB (MATLAB and Statistics Toolbox Release 2018a, The Mathworks, Inc., Natick, Massachusetts, United States). Pre-processing steps were as follows: (a) correcting slice-timing; (b) motion correction by realigning images; (b) reorienting structural and functional images manually; (c) co-registering structural images into functional images and segmenting to gray matter, white matter, and cerebrospinal fluid; (d) reducing potential influences of motion-related artifacts from functional data through (i) regressing out 24 Friston-model head-motion estimates and nuisance covariates (white matter and cerebrospinal fluid signals) (42) and (ii) scrubbing outlier volumes with mean framewise displacement (FD) >0.2 mm using spline interpolation (19, 43, 44); and (e) normalizing functional images to Montreal Neurological Institute standard space by Diffeomorphic Anatomical Registration Through Exponentiated Lie algebra Method (45) and reslicing to 3.0 × 3.0 × 3.0 mm^3^.

### Controlling for Other Confounding Signals

(a) Noises associated with machine instability were minimized by removing the first 10 image volumes prior to pre-processing, resulting in 230 volumes. (b) Low-frequency drifts and high-frequency aliasing were band-pass filtered (0.01–0.08 Hz) after data normalization (d). Only data with maximum translation <1 mm and rotation <1.0 degree were included for further analysis. With respect to these confounding factors, further analyses were performed with 16 HIV patients and 16 HCs.

### Group-Level Analysis for Confounding Variables: Age and Mean FD

To test whether confounding variables (age and mean FD) present significant differences between the groups, two-sample *t*-tests were performed using SPSS software [IBM SPSS Statistics for Windows, version 20.0 (IBM Corp., Armonk, N.Y., USA)]. No significant differences in age (*p* > 0.05; Table 1) was observed; however, HIV patients had a relatively lower mean FD (*p* < 0.05) compared with controls.

### Defining Regions of Interest, Network Construction, and Static RSFC Analysis

The brain networks were constructed from 90 ROIs defined in an automated anatomical labeling (AAL-90) atlas (46). The AAL-90 atlas does not include the cerebellum and is the most popular atlas widely used to identify brain changes in recent years (47). The regions in the networks were partitioned according to their locations in the brain, yielding six subnetworks, namely prefrontal, frontal, parietal, occipital, temporal, and subcortical subnetworks (48). To test whether there is an intersection between the static and dynamic RSFC or whether they are supplemental to each other in understanding the neural mechanisms underlying early cognitive impairment in HIV, we computed static z-transformed Pearson's correlations over a full range of time (230-image volumes).

### Dynamic RSFC and Its Temporal Properties Analysis

For dynamic RSFC analyses, we first constructed dynamic correlation maps by sliding a window (WL = 30 s) to 230-TR fMRI data (data length, 460 s), conforming to the procedures described by Allen et al. (25). The window was designed to detect a wide range of transitions as described in the previous study (39). The proposed window length (WL = 30 s) can separate cognitive process-specific dynamics while keeping better correlation estimates (37, 49). Having slid the window by the 2-s step size (1TR) (25), 216 (230 – 15 + 1) windowed correlation maps were generated for each subject. Two hundred and sixteen Fisher's z-transformed correlation maps (90 × 90 matrices) were produced, regarded as dynamic RSFC maps.

**Figure 1:**
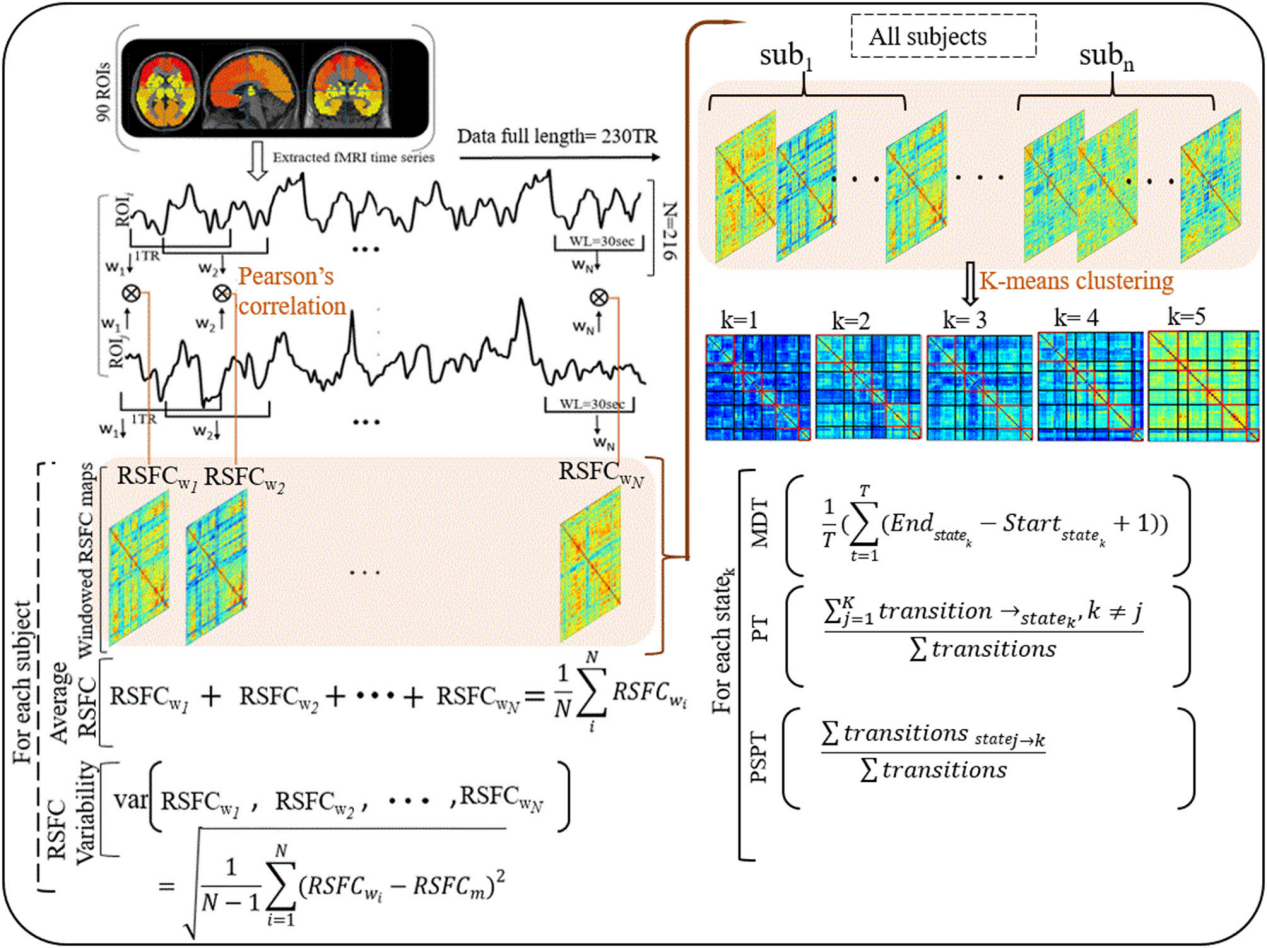
Illustrations of procedures for dynamic RSFC and state analyses. Ninty ROIs' times series were extracted from fMRI data (230 TR) and sliding windows (WL = 30 s) were used to generate dynamic RSFC maps. Dynamic RSFC properties (average dynamic RSFC, and connectivity variability) were computed across windows. Optimal clusters (states, *k* = 1–5) were generated using K-means clustering. State properties [the mean dwelling time of the state (MDT), and state transitioning (PT, PSPT)] were further estimated from each state. RSFC_wi_ represents dynamic RSFC map at window w_*i*_; MDT and PT denote the mean dwelling and probability of transitioning to state_*k*_ respectively, whereas PSPT signifies the probability of specific transitioning from state_*j*_ to state_*k*_.

To understand how properties of dynamic RSFC behave in HIV individuals relative to healthy controls, we evaluated the temporal variability (RSFC-SD) and strength (RSFC-STR) of the dynamic RSFC (28, 50, 51). Thus, for each subject, the temporal variability of dynamic RSFC was obtained by computing the standard deviation of each ROI–ROI correlation pair across all dynamic windows (*N* = 216), reflecting the dynamic reconfiguration of a brain into distinct functional modules at a different time and is indicative of brain flexibility and adaptability (52, 53). The strength of dynamic RSFC was estimated by averaging ROI–ROI correlation pairs across all windows (*N* = 216) (Figure 1 and Supplementary Table 3).

### Dynamic RSFC State Clustering Analysis

Then, we adopted a k-means algorithm with L1 distance function to estimate the dominant recurrent patterns—regarded as states—of the dynamic RSFC (25, 31, 32). From 216 windows × 4,005 features of each subject, we identified windowed covariance matrices (exemplars) with local maxima to use for clustering initialization (54). This reduces redundancy between windows and computational load during clustering (25). One thousand and sixty-four exemplars were identified and adopted for initial clustering with 500 repetitions to escape local minima (55). The resulting centroids were used to initialize clustering for all data (32 participants × 216 windows = 6,912 instances). Similar to Abrol et al. (54), we estimated the valid optimal number of clusters (from *k* = 2–20) using the cluster validity index based on the elbow criterion (54). The resulting cluster medians were regarded as the dominant RSFC states. The reproducibility of these states was in accordance with Abrol et al. (54) and Allen et al.'s (25) studies (25, 54).

### Characterization of Dynamic RSFC States' Properties

To investigate the effects that HIV may have on the properties of transient states, we examined three state properties—mean dwelling time, state transitioning, and state network topology. The mean dwelling time measured by the metric MDT reflects the time an individual spends in a particular state (27, 51, 56, 57). State transitioning measured by the metric PT denotes the probability of transitioning to a particular state *i* from other states (*i* ≠ *k*) (33, 58, 59). Further, we extended characterizing state transitioning by quantifying the transition from specific state *j* to specific state *k* (*j* ≠ *k*), measured by the metric PSPT (the likelihood of the state *j* to transit to state *k* and not otherwise) (Figure 1 and Supplementary Table 3). Network configurations of the states demonstrating alterations in MDT, PT, or PSPT were further assessed (31, 33, 60).

### Statistical Analyses

The general linear model (GLM) examined the differences in static and dynamic RSFC between HIV patients and healthy controls. Results were further false discovery rate-corrected (*p* < 0.05, FDR) with age and mean FD as covariates using the Gretna toolbox (61). Permutation tests (*p* < 0.05) evaluated the differences between groups in state properties (MDT, PT, and PSPT) (56). Pearson's correlations were performed to examine the relationship between imaging markers and clinical measures using SPSS software, controlling for age, and mean FD (*p* < 0.05). The relationship between altered properties of RSFC and states was further assessed.

## Results

### Static and Dynamic RSFC Group Differences

**Figure 2:**
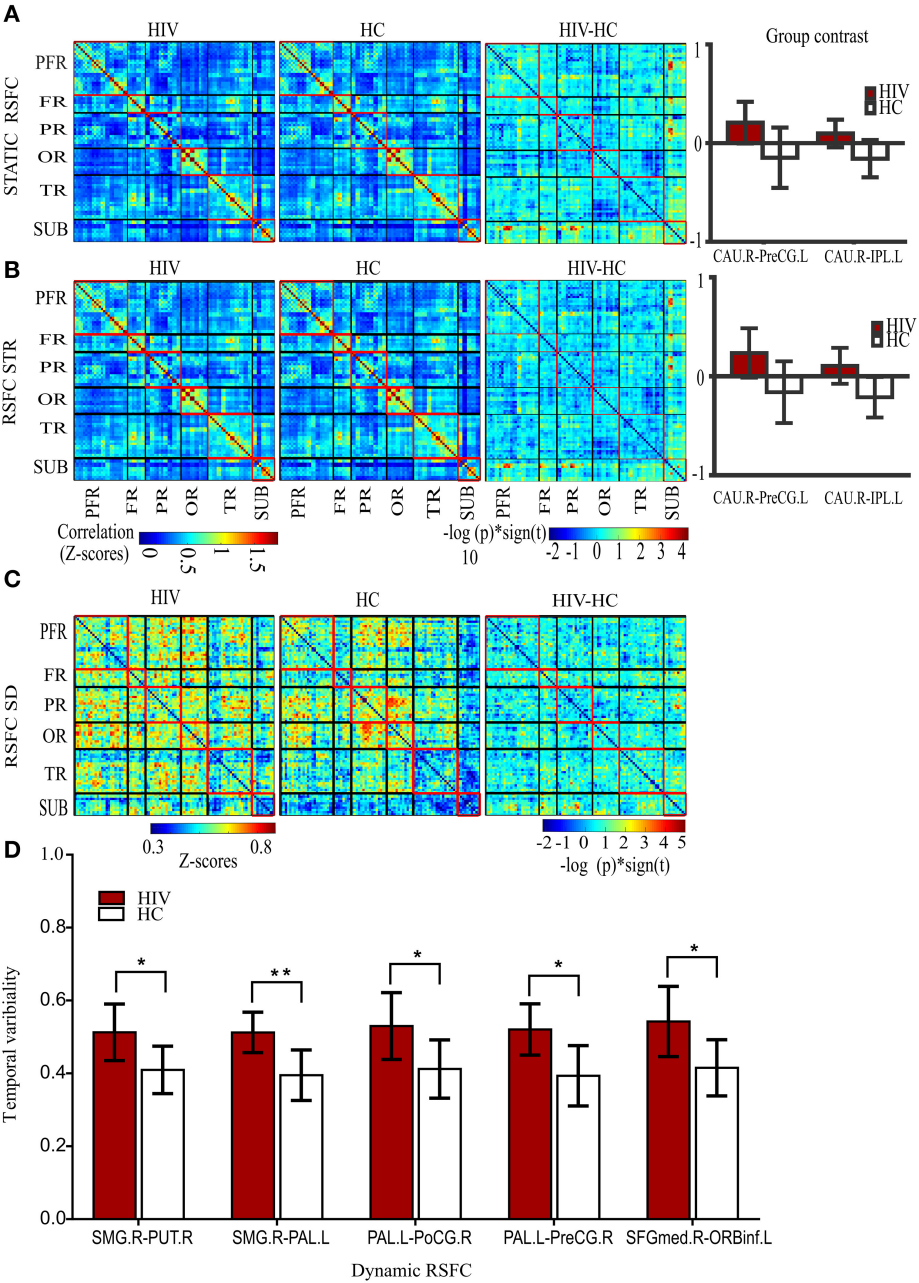
Static and dynamic functional connectivity. **(A)** The static RSFC maps for HIV and HC groups, group difference map (HIV-HC), and between-group contrast. **(B)** The average dynamic RSFC maps for HIV and HC groups, group difference maps (HIV-HC), and between-group contrast in average dynamic RSFC. **(C)** The mean dynamic temporal variability maps for HIV and HC groups, and group variability-difference maps (HIV-HC). **(D)** The group difference for temporal variability as depicted in the histogram. PFR, prefrontal regions; FR, other frontal regions; PR, parietal regions; OR, occipital regions; TR, parietal regions; and SUB, subcortical regions. R, right; L, left; CAU, the caudate nucleus; IPL, inferior frontal lobe; and PreCG, precentral gyrus; SMG, supramarginal gyrus, PUT, putamen; PAL, pallidum; PoCG, postcentral gyrus; SFGmed; superior frontal (medial) gyrus; ORBinf, the pars of orbitalis of the inferior frontal gyrus. Pearson's correlation coefficients were all fisher's z-transformed. The group-difference (HIV-HC) connectivity maps are displayed as –log10 (*p*-value) × sign (*t*).

Our results showed significantly increased static RSFC between the right caudate nucleus (CAU.R) and left pre-central gyrus (PreG.L), and between the right caudate nucleus (CAU.R) and left inferior parietal gyrus (IPL.L) in HIV patients (Figure 2A and Table 2). The rest of the regions did not report significant differences in static RSFC between the groups.

**Table 2:** Group analysis results of static and dynamic functional connectivity.

**Functional pairs**	***T*-value**	***P*-value**
**Static RSFC**		
CAU.R-PreCG.L	3.83	0.000609
CAU.R-IPL.L	4.35	0.000146
**Average dynamic (RSCF-STR)**		
CAU.R-PreCG.L	3.97	0.000414
CAU.R-IPL.L	4.65	0.000063
**Temporal variability (RSCF-SD)**		
SMG.R-PUT.R	4.06	0.000324
SMG.R-PAL.L	5.30	0.000009
PAL.L-PreCG.R	4.67	0.000591
PAL.L-PoCG.R	3.88	0.000531
SFGmed.R-ORBinf.L	4.12	0.000274

*P-values were computed based on two-sample t-test. R, right; L, left; CAU, the caudate nucleus; PreCG, precentral gyrus; IPL, inferior parietal lobe; SMG, supramarginal gyrus; PAL, pallidum; PUT, putamen; PoCG.R, postcentral gyrus; SFGmed, superior frontal(medial) gyrus; ORBinf, the pars of orbitalis of the inferior frontal gyrus*.

A significant increase in the strength (average) of dynamic RSFC was also reported between the right caudate nucleus and left pre-central gyrus, and between the right caudate nucleus and left inferior parietal gyrus in HIV patients, compared to healthy controls (Figure 2B and Table 2). The average dynamic RSFC of other regions did not reach significance.

Compared to healthy controls, temporal variability of dynamic RSFC in HIV patients was greater between the right supramarginal gyrus (SMG.R) and right putamen; the right SMG and left pallidum; the left pallidum and right post-central gyrus; the left pallidum (PAL.L) and right pre-central gyrus (PreCG.R); and the right superior frontal (medial) gyrus (SFGmed.R) and pars orbitalis of the left inferior frontal gyrus (ORBinf.L) (Figures 2C,D and Table 2). No significant differences were observed in the dynamic RSFC variability of other functional connections.

### Group Differences in Dynamic RSFC States

**Figure 3:**
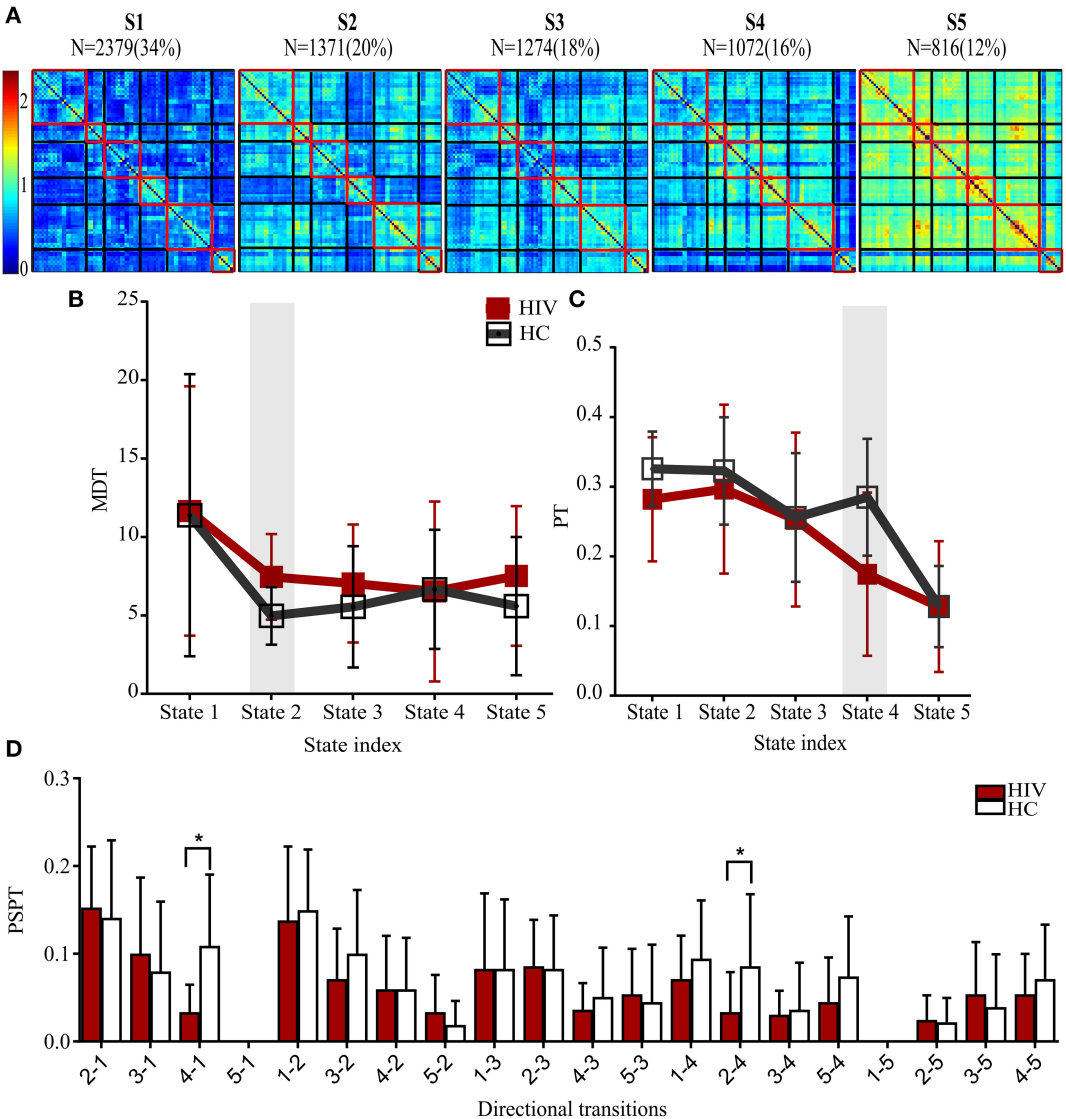
States properties of HIV infected individuals and healthy controls. **(A)** Five transient states generated by K-means clustering analysis, displayed with the number of correlation maps constituting to the state. **(B)** Mean dwelling time (MDT) between groups. **(C)** The probabilities of transitioning (PT) for each state in HIV and HC groups. **(D)** The probabilities of specific state-to-state transitioning (PSPT). On top of the figures are the numbers (*N*=) of dynamic correlation maps [with their percentages (%)] showing the population of correlation maps assigned to those states during clustering. Group differences (HIV-HC) were significant at *p* < 0.05.

States and their features are shown in Table 3. The clustering analyses suggested five transient states, State 1 (S1) with 34% (*N* = 2,379) of the proportion of dynamic correlation maps, State 2 (S2) with 20% (*N* = 1,371), State 3 (S3) with 18% (*N* = 1,274), State 4 (S4) with 16% (*N* = 1,072), and State 5 (S5) with 12% (*N* = 816). State 1 and state 5 were characterized by globally hypo-connected and hyper-connected patterns, respectively. State 2 was typified by weaker occipital connectivity. State 3 had frontal and prefrontal hypo-connectivity. State 4 was associated with weaker subcortical connectivity (Figure 3). Compared with controls, HIV patients had lower transitions (PT) to state 4. Reduced state transitioning (PSPT) from state 4 to state 1 and from state 2 to state 4 was also observed. Furthermore, we found that patients with HIV had higher dwelling time (MDT) in state 2 compared with healthy controls (Figure 3 and Table 3).

**Table 3:** Group difference in state properties between HIV and HC groups and categorization of state network connectivity.

**State properties**	***T*-value**	***P*-value**
MDT (state 2)	3.03	0.0050
PT (state 4)	−2.25	0.0317
PSPT (state 4-state 1)	−3.40	0.0019
PSPT (state 2-state 4)	−2.18	0.0372
**States**	**Network connectivity**	**Percentage (hypo, %**)
State 1	Globally hypo-connected	63
State 2	Occipitally hypo-connected	20
State 3	Fronto-parietally hypo-connected	21
State 4	Subcortically hypo-connected	18
State 5	Globally hyperconnected	2

*P-values were computed based on permutation test. MDT, mean dwelling time; PT, probability of transitioning to a state_i_; PSPT, probability of transitioning from state_i_ to state_j_; the criteria for categorizing state network connectivity was based on the proportions of connectivity type observed (Z < 0.25)*.

### Relationship Between Imaging Markers and Clinical Characteristics

**Figure 4:**
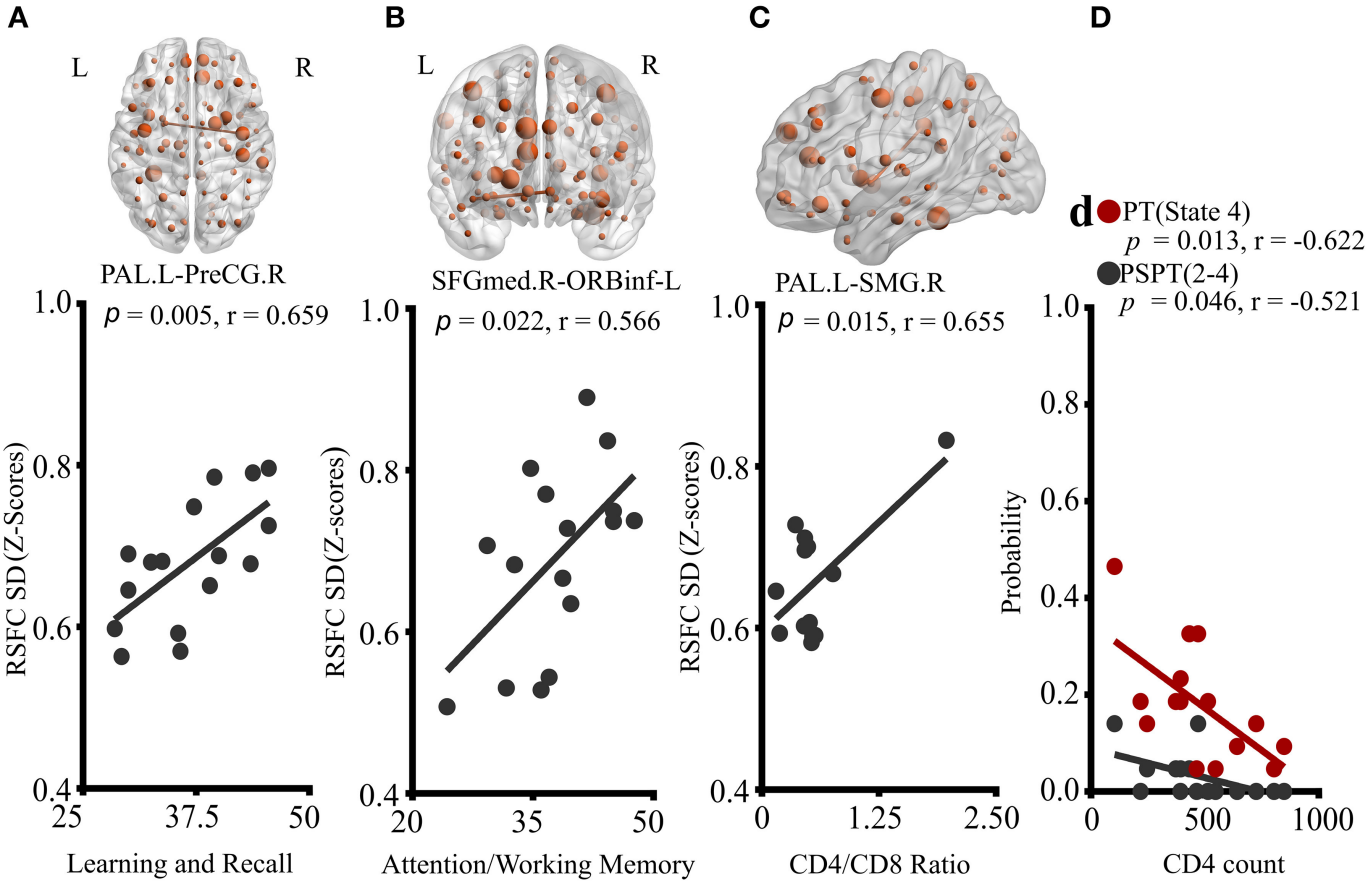
Correlations of properties of dynamic RSFC and RSFC states with clinical characteristics of HIV patients. **(A)** Correlation of PAL.L-to-PreCG.R temporal variability with learning and recall score. **(B)** Association of temporal variability of SFGmed.R-to-ORBinf-L with Attention/working memory performance. **(C)** The relationship between PAL.L-SMG.R variability and CD4^+^/CD8^+^ ratio. **(D)** Relationships of probabilities of transitioning to state 4, and state-2-to-state-4 transitioning with CD4^+^ T-cell counts. The displayed relationships were evaluated in Pearson's correlations coefficients, *r*; *p* < 0.05. *P, p*-value; L, left; R, right; PAL, the pallidum; PreCG, precentral gyrus; SFGmed, superior frontal (medial) gyrus; ORBinf, pars orbitalis of the inferior frontal gyrus; and SMG, supramarginal gyrus.

Correlation results are reported in Figure 4. Neither neuropsychological results nor blood results were correlated with static RSFC. However, in dynamic RSFC analyses, altered dynamic RSFC variability and cognitive performances revealed significant relationships. Specifically, greater dynamic RSFC variability of PAL.L-PreCG.R showed a positive relationship with cognitive Leaning and Recalling (memory) (Figure 4A; *P* = 0.005, *r* = 0.659). We also observed a strong association between the dynamic variability of SFGmed.R-ORBinf.L and cognitive attention/working memory (Figure 4B; *P* = 0.022, *r* = 0.566). While the variability of PAL.L-SMG.R was related to CD4+/CD8+ ratio (Figure 4C; *P* = 0.015, *r* = 0.655), reduced state transitioning and CD4+ T-cell counts were negatively correlated (Figure 4D; PT, state 4, *P* = 0.013, *r* = −0.622; PSPT, state 2-to-state 4, *P* = 0.046, *r* = −0.521).

### Relationship of State Properties and Static and Dynamic RSFC

In HIV patients, fewer transitions (PT) to state 4 demonstrated strong correlations with altered static RSFC of CAU.R-to-PreCG.L (*P* = 0.003, *r* = −0.686) and CAU.R-IPL.L (*P* = 0.031, *r* = −0.518; Supplementary Figure 3A). A similar relationship existed between PT of state 4 and average dynamic RSFC (STR) of CAU.R-to-PreCG.L (Supplementary Figure 3B; *P* = 0.026, *r* = −0.553). Results also showed that higher mean dwelling time (MDT) of state 2 was related to average dynamic RSFC of CAU.R-IPL.L (Supplementary Figure 3C; *P* = 0.031, *r* = 0.540).

### Robustness of the Results

To test the reproducibility of these results at 30–60 s window sizes, we examined properties of dynamic RSFC across different window sizes [30 s (15TR), 44 s (22TR), and 60 s (30TR)]. We found that results of both average dynamic RSFC (Supplementary Figure 1) and temporal variability (Supplementary Figure 2) were reproducible within the specified range of sliding window size, consistent with previous empirical studies (29, 32, 37, 49). We also assessed the consistency of our results using network-based statistics (FDR, corrected) (62) and found that our findings were also consistent.

## Discussion

The present study investigated the dynamic aspects of RSFC, providing insight into understanding HIV pathology. We presented methodological improvements designed to capture low-level state-to-state transitions. To the best of our knowledge, this study is the first to explore the dynamic functional connectivity in HIV patients using fMRI data.

### Spatial Overlap Between Static RSFC and Average Dynamic RSFC

Increased caudate functional connectivity to pre-central and inferior parietal regions observed in both static and dynamic (average) RSFC suggests that average dynamic RSFC estimated across windows complement to static RSFC estimated over the whole BOLD signals over time. Moreover, these results may signify that static RSFC can be deduced from dynamic RSFC and that dynamic RSFC provides a broader characterization of functional changes. Several previous cross-sectional studies have suggested that the caudate nucleus and the primary sensorimotor, pre-motor, and inferior parietal cortices are selectively vulnerable in HIV disease (12, 63, 64). It is worth noting that the caudate nucleus and primary sensorimotor cortex integrate during motor control and learning functioning; thus, the observed alterations in caudate-to-pre-central connectivity suggest a possible HIV-associated brain injury that might impair spatial-motor memory and learning (12, 64). Previous studies have also detected neural infiltration of the inferior parietal lobe in the caudate nucleus (65), implying the existence of communication between these regions. Melrose et al. (66) found that this communication is susceptible to HIV infection. For example, during semantic event sequencing, patients with HIV exhibited greater caudate-to-parietal connectivity (66). Our results demonstrating increased caudate-parietal connectivity support these studies that disrupted communication between subsystems of these regions is attributed to HIV infection. Since neurons of the brain are multimodal (involved in differential brain functions) (67–70), increases in caudate connectivity to IPL and pre-central gyrus may reflect compensatory plasticity necessitated to retain their functions following HIV injury.

### Higher Temporal Striato-Sensorimotor Dynamic RSFC Variability and Learning and Recall

Previous studies have demonstrated how brain flexibility and plasticity are important for learning ability, gene expression, and fluid intelligence (29, 32, 71). The brain reconfigures its functional interactions or circuitry over time while learning, a property which is vulnerable to injuries, neurological disease, psychiatric disorders, or cognitive aging (72). Our results illustrating increasing striato-sensorimotor variability with cognitive learning and recall may suggest (i) the striato-sensorimotor connectivity and its flexibility are relevant to human learning and recall. Thus, it can be used for the prediction of learning capacity. This hypothesis could be supported by previous studies that demonstrated how these regions play an important role in early, long-term, and categorical learning or that linked the dynamics of these regions with learning stage (early, middle, or late) predictions (72–77). (ii) These findings also suggest that striato-sensorimotor circuitry may be one of the primary targets of HIV infection, consistent to other previous studies (15). (iii) Moreover, altered striato-sensorimotor dynamics might be a functional basis for cognitive impairments, with the hypothesis that intensively lowered variability reflecting severe learning deficits. (iv) Increases in striato-sensorimotor temporal variability in patients on cART might be a sign of brain compensatory mechanisms or cART-necessitated CN immune improvements triggering plasticity to restore or preserve brain functions. This can also be supported by earlier studies showing that cART improves neural functions (20), and those increasing dynamics are likely to occur after rehabilitation to restore brain functionality (32), or in response to cognitive demands (78).

### Higher Frontal Dynamic RSFC Variability and Attention and Working Memory

Our findings demonstrating greater dynamic RSFC variability between the pars orbitalis of the left inferior frontal and right frontal (medial) gyri related to attention and working memory in HIV patients may point to the fact that brain flexibility may not be limited to functions such as injury recovery, adaptive responses, or a growth mindset (79–82) but rather extends to a wide range of roles, including attention and working memory. PFC-system reconfigurations may be required for capturing temporal storage and active manipulation of the stored information (78). Frontal regions' participation in attention and working memory has already been explained previously (83–85). Studies have reported that the execution of attention and working memory highly requires the flexibility of frontal and or frontal-related frontal systems than non-frontal systems (78). This may be the case since the lateral prefrontal cortices (ventrolateral PFC and dorsolateral PFC) are functionally and anatomically connected through arcuate fibers (86, 87), becoming critical nodes that work together for a variety of cognitive functions (87). They are also connected to the executive network and DMN dorsolaterally to control for the dynamic allocation of attention (88). Furthermore, increases in frontal variability observed in HIV patients suggest attenuations HIV might have in temporal reconfigurations of these PFC circuits, agreeing with previous HIV studies demonstrating attenuated activation in PFC during complex attention, and working memory tasks in infected male patients (7, 89).

### Greater Striato-Parietal Dynamic Variability and HIV-Laboratory Markers

Greater variability found in the dynamic RSFC between supramarginal and regions of striatum (putamen and pallidum) also suggests abnormal patterns associated with HIV. This could be supported by the observed strong correlation (especially, in PAL.L-SMG.R) with the CD4+/CD8+ ratio. This result confirms the evidence derived from static RSFC studies that HIV pathology includes impaired communication between striato-parietal regions (20, 22, 90–92), consistent with our static results of attenuated caudate–IPL connectivity. The positive relationship between variability and CD4+/CD8+ ratio may also indicate the ability of temporal variability to predict the progress of HIV in the brain or the decline of CNs immune systems. Given that the CD4+/CD8+ ratio is a more accurate hallmark of the status of the body immune system (93, 94), we speculate that monitoring of trends of the CN CD4+/CD8+ ratio balance on differential antiretroviral therapies can be achieved through tracking the temporal variability, supporting earlier studies detecting functional changes with switching antiretroviral drugs (17). Again, since cART repairs innate CNs by reducing inflammation and enhances synoptodentritic plasticity that improves dynamic synaptic communication and neural functions (4, 95), we hypothesize that the observed variability increases in HIV patients on stable cART might also be attributed to cART stabilization; however, further evidence is required to confirm this supposition.

### Dynamic RSFC States' Properties and Their Relationship With an HIV Biomarker

In this study, we observed five hypo/hyper-connected states suggestive of large-scale brain networks being characterized by hypo/hyper-connected patterns, consistent with other dynamic RSFC studies (31, 32, 96). In contrast to a study by Mash et al. (52) who considered clustering the data of patients and healthy controls separately, this study did not cluster data separately. This conformed to a large body of earlier studies (33, 56). It is worth noting that clustering the groups separately could result in mismatched clusters (52). The maximum whole correlation approach often used to estimate the paring clusters is also likely to match two or more clusters to a cluster of another group (52), which would affect the analyses. Furthermore, we hypothesize that clustering groups separately influence group differences fundamentally as clustering foundations such as local minima's are data-dependent, which are likely to be different between groups.

As noted above, state 2 was typified by weaker connectivity within occipital networks, paralleled by a longer dwelling time pattern of the state. Several studies of HIV have identified structural and functional abnormalities in the occipital lobe, suggesting the presence of HIV-associated injuries (3, 12, 97–101). The cortical thickness of the occipital cortex is one of the major domains, which is progressively affected by HIV (12, 101). The neurons of the visual cortex of the occipital lobe are also known to undergo atrophy in HIV patients (98, 99). A study by R. Li et al. found a deficiency in spontaneous activity of the occipital lobe in early HIV infection (100). While a dysfunction of the occipital lobe was not clinically implicated in HAND in earlier studies, sufficient evidence implicated HIV in gray matter loss, supported by their associations to CD4+ T-cell counts (102). Later studies revealed a link between impaired spontaneous activity in the occipital cortex and deficits in executive function or CD4+/CD8 ratio (100). The clinical picture of the relationship between HAND and deficits in the occipital lobe in patients without and with cART is increasingly becoming clear. Emerging evidence suggests that generalized degradation of gray matter, disruption of spontaneous activity, and attenuation of functional activation and connectivity, involving the occipital cortex, could explain deficits seen in visual attention and visuospatial processing in patients with HIV (3, 89). Our observed changes in temporal properties of dynamic RSFC which depict altered dwelling time in states with weaker occipital connectivity may be related to an initial dysfunction of the occipital cortex and are in support of the above hypothesis. In other aspect, increased amount of time spent could be interpreted as a potential compensatory mechanism of the brain networks resulting in prolonged stay in occipitally hypoconnected state.

In HIV patients, state transitioning (PT) of state 4 typified by weaker connectivity in subcortical networks was reduced, paralleled by reduced switching (PSPT) from state 4 to state 1 and from state 2 to state 4. Regions of the subcortical networks are susceptible to HIV (20). Among other effects, gray matter volume loss, activity modulation loss, subcortical network integration loss have been reported (10, 19). The reduction of state transitioning of the state with weaker subcortical connectivity (state 4) was observed to be negatively correlated with CD4+T-cell counts, possibly indicating an association between HIV severity and switching between states. Reduced switching from state 2 to state 4 also presented a similar pattern of association to HIV severity, a finding that can only be detected precisely at low-level of state transitioning analyses. HIV has been postulated to have varying effects on the brain (103). Therefore, among the possibilities of this reversed relationship could be evidence of such variant effects of HIV or a potential compensatory mechanism resulting in a somewhat increasing state transitioning as CD4+ T-cell counts deteriorate following HIV and cART. A similar phenomenon was previously reported (104, 105) between CD4+T-cell counts and static RSFC.

One would like to relate our observed results across diseases. Among the findings worth noting is that while HIV patients appeared to spend a longer time in states typified by weaker connectivity in occipital networks, parallel to patients with schizophrenia (33, 35), patients with Parkinson's disease spent a shorter time in states with weaker connectivity (31). Findings showing patients with schizophrenia spending a shorter time in states with strong connectivity in global networks are inconsistent with those we found in HIV patients, as neither MDT nor PT of globally hyperconnected states (state 5) presented differences between groups. This may suggest that abnormal patterns related to HIV are associated with region-specific network alterations—such as subcortical and occipital regions alterations (3, 12, 97–101)—rather than global network alterations.

### Correlations of State Properties With Dynamic RSFC Properties

We noticed that the mean dwelling time of the state with weaker connectivity in occipital networks was related to the average dynamic RSFC of the caudate nucleus and regions of the left inferior parietal lobe. Moreover, reduced state transitioning of the state with weaker subcortical connectivity also presented a strong relationship with both static and average dynamic RSFC. Together, these findings demonstrate that state properties may supplement both static and dynamic RSFC in delineate HIV pathology, expanding knowledge to the existing literature.

### Limitation

The present study presents some limitations. Although the number of samples might be relatively larger compared with previous studies of HIV (7, 89), large cohorts are required to validate these findings. This study used data from male patients; this may be common in some of the HIV studies (7, 89, 106) as HIV data availability is challenging. The design of our study was based on the hypothesis that HIV can lead to cognitive dysfunction. Thus, the assessment of cognitive performances did not include healthy controls, consistent with earlier studies (100, 101). However, the lack of neuropsychological tests for healthy controls may also limit the study. It is also important to note that the current study is significant for the majority of the population living with HIV nowadays. Nonetheless, other less represented groups in the HIV+ population worldwide should be assessed in future studies. Both static RSFC and dynamic RSFC analyses were performed using ROI pairs of the whole brain regions defined by AAL-90 atlas, future studies are required to validate these findings with data-driven approaches. In correlation analyses, we did not correct for multiple comparisons, in line with other studies (3, 15). We also suggest that future studies should include gender-matched cohorts.

## Conclusion

In the present study, we applied dynamic resting-state functional connectivity analyses to investigate dynamic alterations that may underlie early cognitive impairments in HIV patients on cART. We found that HIV patients exhibit greater temporal variability related to learning, attention and working memory, and CD4+/CD8+ ratio. HIV patients also appear to have reduced state transitioning associated with CD4+T cell counts. We conclude that HIV affects brain connectivity dynamics and that these abnormal dynamic patterns may contribute to persisting cognitive impairments in patients with HIV. These findings also indicate that dynamic RSFC analyses may provide an impetus for detecting other pathological dimensions of HIV on the brain in patients receiving antiretroviral therapy.

## Data Availability Statement

The raw data supporting the conclusions of this article will be made available by the authors, without undue reservation.

## Ethics Statement

The studies involving human participants were reviewed and approved by the Ethical Committee of the Capital Medical University and the University of Science and Technology of China. The patients/participants provided their written informed consent to participate in this study.

## Author Contributions

BN, JZ, YL, and XW substantial contributions to the conception or design of the work. BN, JZ, YWa, YWe, JU, and HL contributions to the acquisition, analysis or interpretation of data. BN, XW, HL, and BQ drafting the work or revising it critically for important intellectual content. HL and BQ final approval of the version submitted. All authors contributed to the article and approved the submitted version.

## Conflict of Interest

The authors declare that the research was conducted in the absence of any commercial or financial relationships that could be construed as a potential conflict of interest.

## References

[1] Abidin AZ Dsouza AM Nagarajan MB Wang L Qiu X Schifitto G. Alteration of brain network topology in HIV-associated neurocognitive disorder: a novel functional connectivity perspective. Neuroimage Clin. (2018) 17:768–777. 10.1016/j.nicl.2017.11.025 29527484 PMC5842750

[2] Antinori A Arendt G Becker JT Brew BJ Byrd DA Cherner M. Updated research nosology for HIV-associated neurocognitive disorders. Neurology. (2007) 69:1789–99. 10.1212/01.WNL.0000287431.88658.8b 17914061 PMC4472366

[3] Wiesman AI O'Neill J Mills MS Robertson KR Fox HS Swindells S. Aberrant occipital dynamics differentiate HIV-infected patients with and without cognitive impairment. Brain. (2018) 141:1678–90. 10.1093/brain/awy097 29672678 PMC5972635

[4] Ellis R Langford D Masliah E. HIV and antiretroviral therapy in the brain: neuronal injury and repair. Nat Rev Neurosci. (2007) 8:33–44. 10.1038/nrn2040 17180161

[5] McIntosh RC Rosselli M Uddin LQ Antoni M. Neuropathological sequelae of human immunodeficiency virus and apathy: a review of neuropsychological and neuroimaging studies. Neurosci Biobehav Rev. (2015) 55:147–64. 10.1016/j.neubiorev.2015.04.008 25944459

[6] Lew BJ McDermott TJ Wiesman AI O'Neill J Mills MS Robertson KR. Neural dynamics of selective attention deficits in HIV-associated neurocognitive disorder. Neurology. (2018) 91:E1860–9. 10.1212/WNL.0000000000006504 30333162 PMC6260195

[7] Ernst T Chang L Jovicich J Ames N Arnold S. Abnormal brain activation on functional MRI in cognitively asymptomatic HIV patients. Neurology. (2002) 59:1343–49. 10.1212/01.WNL.0000031811.45569.B0 12427881

[8] Janssen MA Meulenbroek O Steens SC Góraj B Bosch M Koopmans PP. Cognitive functioning, wellbeing and brain correlates in HIV-1 infected patients on long-term combination antiretroviral therapy. Aids. (2015) 29:2139–48. 10.1097/QAD.0000000000000824 26544578

[9] Zhao J Chen F Ren M Li L Li A Jing B. Low-frequency fluctuation characteristics in rhesus macaques with SIV infection: a resting-state fMRI study. J Neurovirol. (2019) 25:141–9. 10.1007/s13365-018-0694-5 30478797

[10] Aylward EH Hendere JD McArthur JC Brettschneider PD Harris GJ Barta PE. Reduced basal ganglia volume in HIV-1-associated dementia-Results. J Neurol. (1993) 43:2099–104. 10.1212/WNL.43.10.2099 8413973

[11] Ragin AB Du H Ochs R Wu Y Sammet CL Shoukry A. Structural brain alterations can be detected early in HIV infection. Neurology. (2012) 79:2328–34. 10.1212/WNL.0b013e318278b5b4 23197750 PMC3578377

[12] Thompson PM Dutton RA Hayashi KM Toga AW Lopez OL. Thinning of the cerebral cortex visualized in HIV/AIDS reflects CD4^+^ T lymphocyte decline. Proc Natl Acad Sci. (2005) 102:15647–52. 10.1073/pnas.0502548102 16227428 PMC1266080

[13] Heaps JM Sithinamsuwan P Paul R Lerdlum S Pothisri M Clifford D. Association between brain volumes and HAND in cART-naive HIV+ individuals from Thailand. J Neurovirol. (2015) 21:105–12. 10.1007/s13365-014-0309-8 25604494 PMC4375016

[14] Thompson PM Jahanshad N. Novel neuroimaging methods to understand how HIV affects the brain. Curr Hiv/Aids Rep. (2015) 12:289–98. 10.1007/s11904-015-0268-6 25902966 PMC4433315

[15] Yadav SK Gupta RK Hashem S Bhat AA Garg RK Venkatesh V. Changes in resting-state functional brain activity are associated with waning cognitive functions in HIV-infected children. Neuroimage Clin. (2018) 20:1204–10. 10.1016/j.nicl.2018.10.028 30391858 PMC6224323

[16] Zhao J Jing B Chen F Liu J Wang Y Li H. Altered regional homogeneity of brain spontaneous signals in SIV infected rhesus macaque model. Magn Reson Imaging. (2017) 37:56–61. 10.1016/j.mri.2016.10.019 27989909

[17] Toniolo S Cercignani M Mora-Peris B Underwood J Alagaratnam J Bozzali M. Changes in functional connectivity in people with HIV switching antiretroviral therapy. J Neurovirol. (2020) 26:754–63. 10.1007/s13365-020-00853-0 32500477 PMC7532134

[18] Zang YF Jiang TZ Lu YL He Y Tian LX. Regional homogeneity approach to fMRI data analysis. Neuroimage. (2004) 22:394–400. 10.1016/j.neuroimage.2003.12.030 15110032

[19] Ipser JC Brown GG Bischoff-Grethe A Connolly CG Ellis RJ Heaton RK. HIV infection is associated with attenuated frontostriatal intrinsic connectivity: a preliminary study. J Int Neuropsychol Soc. (2015) 21:203–13. 10.1017/S1355617715000156 25824201 PMC4400233

[20] Ortega M Brier MR Ances BM Effects of HIV and combination antiretroviral therapy on cortico-striatal functional connectivity. Aids. (2015) 29:703–12. 10.1097/QAD.0000000000000611 25849834 PMC4391231

[21] McIntosh RC Chow DC Lum CJ Hidalgo M Shikuma CM Kallianpur KJ Reduced functional connectivity between ventromedial prefrontal cortex and insula relates to longer corrected QT interval in HIV plus and HIV- individuals. Clin Neurophysiol. (2017) 128:1839–50. 10.1016/j.clinph.2017.07.398 28826014 PMC5612780

[22] Samboju V Philippi CL Chan P Cobigo Y Fletcher JLK Robb M. Structural and functional brain imaging in acute HIV. Neuroimage Clin. (2018) 20:327–35. 10.1016/j.nicl.2018.07.024 30101063 PMC6082997

[23] Chaganti JR Heinecke A Gates TM Moffat KJ Brew BJ. Functional connectivity in virally suppressed patients with HIV-associated neurocognitive disorder: a resting-state analysis. Am J Neuroradiol. (2017) 38:1623–9. 10.3174/ajnr.A5246 28596187 PMC7960403

[24] Egbert AR Biswal B Karunakaran KD Pluta A Wolak T Rao S. HIV infection across aging: Synergistic effects on intrinsic functional connectivity of the brain. Prog Neuro Psychopharmacol Biol Psychiatry. (2019) 88:19–30. 10.1016/j.pnpbp.2018.06.006 29906495

[25] Allen EA Damaraju E Plis SM Erhardt EB Eichele T Calhoun VD. Tracking Whole-Brain Connectivity Dynamics in the Resting State. Cereb Cortex. (2014) 24:663–76. 10.1093/cercor/bhs352 23146964 PMC3920766

[26] Beaty RE Benedek M Silvia PJ Schacter DL Creative cognition and brain network dynamics. Trends Cogn Sci. (2016) 20:87–95. 10.1016/j.tics.2015.10.004 26553223 PMC4724474

[27] Calhoun VD Miller R Pearlson G Adali T. The chronnectome: time-varying connectivity networks as the next frontier in fMRI data discovery. Neuron. (2014) 84:262–74. 10.1016/j.neuron.2014.10.015 25374354 PMC4372723

[28] Kaiser RH Whitfield-Gabrieli S Dillon DG Goer F Beltzer M Minkel J. Dynamic resting-state functional connectivity in major depression. Neuropsychopharmacology. (2016) 41:1822–30. 10.1038/npp.2015.352 26632990 PMC4869051

[29] Preti MG Bolton TAW Van De Ville D. The dynamic functional connectome: state-of-the-art and perspectives. Neuroimage. (2017) 160:41–54. 10.1016/j.neuroimage.2016.12.061 28034766

[30] Yao Z Shi J Zhang Z Zheng W Hu T Li Y. Altered dynamic functional connectivity in weakly-connected state in major depressive disorder. Clin Neurophysiol. (2019) 130:2096–104. 10.1016/j.clinph.2019.08.009 31541987

[31] Kim J Criaud M Cho SS Diez-Cirarda M Mihaescu A Coakeley S. Abnormal intrinsic brain functional network dynamics in Parkinson's disease. Brain. (2017) 140:2955–67. 10.1093/brain/awx233 29053835 PMC5841202

[32] Zhang J Cheng W Liu Z Zhang K Lei X Yao Y. Neural, electrophysiological and anatomical basis of brain-network variability and its characteristic changes in mental disorders. Brain. (2016) 139:2307–21. 10.1093/brain/aww143 27421791

[33] Damaraju E Allen EA Belger A Ford JM McEwen S Mathalon DH. Dynamic functional connectivity analysis reveals transient states of dysconnectivity in schizophrenia. Neuroimage Clin. (2014) 5:298–308. 10.1016/j.nicl.2014.07.003 25161896 PMC4141977

[34] Fu Z Tu Y Di X Du Y Pearlson GD Turner JA. Characterizing dynamic amplitude of low-frequency fluctuation and its relationship with dynamic functional connectivity: an application to schizophrenia. Neuroimage. (2018) 180:619–31. 10.1016/j.neuroimage.2017.09.035 28939432 PMC5860934

[35] Du Y Pearlson GD Yu Q He H Lin D Sui J. Interaction among subsystems within default mode network diminished in schizophrenia patients: a dynamic connectivity approach. Schizophr Res. (2016) 170:55–65. 10.1016/j.schres.2015.11.021 26654933 PMC4707124

[36] Wise T Marwood L Perkins AM Herane-Vives A Joules R Lythgoe DJ. Instability of default mode network connectivity in major depression: a two-sample confirmation study. Transl Psychiatry. (2017) 7:e1105. 10.1038/tp.2017.40 28440813 PMC5416685

[37] Xie H Zheng CY Handwerker DA Bandettini PA Calhoun VD Mitra S. Efficacy of different dynamic functional connectivity methods to capture cognitively relevant information. Neuroimage. (2019) 188:502–14. 10.1016/j.neuroimage.2018.12.037 30576850 PMC6401299

[38] Zalesky A Fornito A Cocchi L Gollo LL Breakspear M. Time-resolved resting-state brain networks. Proc Natl Acad Sci USA. (2014) 111:10341–6. 10.1073/pnas.1400181111 24982140 PMC4104861

[39] Shakil S Lee C-H Keilholz SD. Evaluation of sliding window correlation performance for characterizing dynamic functional connectivity and brain states. Neuroimage. (2016) 133:111–28. 10.1016/j.neuroimage.2016.02.074 26952197 PMC4889509

[40] Mbugua KK Holmes MJ Cotton MF Ratai E-M Little F Hess AT. HIV-associated CD4/8 depletion in infancy is associated with neurometabolic reductions in the basal ganglia at age 5 years despite early antiretroviral therapy. AIDS (London England). (2016) 30:1353. 10.1097/QAD.0000000000001082 26959509 PMC4864158

[41] Yan CG Wang XD Zuo XN Zang YF. DPABI: data processing and analysis for (resting-state) brain imaging. Neuroinformatics. (2016) 14:339–51. 10.1007/s12021-016-9299-4 27075850

[42] Friston KJ Williams S Howard R Frackowiak RS Turner R. Movement-related effects in fMRI time-series. Magn Reson Med. (1996) 35:346–55. 10.1002/mrm.1910350312 8699946

[43] Power JD Mitra A Laumann TO Snyder AZ Schlaggar BL Petersen SE. Methods to detect, characterize, and remove motion artifact in resting state fMRI. Neuroimage. (2014) 84:320–41. 10.1016/j.neuroimage.2013.08.048 23994314 PMC3849338

[44] Xie X Cao Z Weng X Jin D Estimating intrinsic dimensionality of fMRI dataset incorporating an AR (1) noise model with cubic spline interpolation. Neurocomputing. (2009) 72:1042–55. 10.1016/j.neucom.2008.04.003

[45] Ashburner J. A fast diffeomorphic image registration algorithm. Neuroimage. (2007) 38:95–113. 10.1016/j.neuroimage.2007.07 17761438

[46] Tzourio-Mazoyer N Landeau B Papathanassiou D Crivello F Etard O Delcroix N. Automated anatomical labeling of activations in SPM using a macroscopic anatomical parcellation of the MNI MRI single-subject brain. Neuroimage. (2002) 15:273–89. 10.1006/nimg.2001 11771995

[47] Long Z Huang J Li B Li Z Li Z Chen H. A comparative atlas-based recognition of mild cognitive impairment with voxel-based morphometry. Front Neurosci. (2018) 12:916. 10.3389/fnins.2018.00916 30574064 PMC6291519

[48] Wang K Liang M Wang L Tian L Zhang X Li K. Altered functional connectivity in early Alzheimer's disease: a resting-state fMRI study. Hum Brain Map. (2007) 28:967–78. 10.1002/hbm.20324 17133390 PMC6871392

[49] Leonardi N Van De Ville D. On spurious and real fluctuations of dynamic functional connectivity during rest. Neuroimage. (2015) 104:430–6. 10.1016/j.neuroimage.2014.09.007 25234118

[50] Kung YC Li CW Chen S Chen SCJ Lo CYZ Lane TJ. Instability of brain connectivity during nonrapid eye movement sleep reflects altered properties of information integration. Hum Brain Map. (2019) 40:3192–202. 10.1002/hbm.24590 30941797 PMC6865651

[51] Liu F Wang Y Li M Wang W Li R Zhang Z. Dynamic functional network connectivity in idiopathic generalized epilepsy with generalized tonic-clonic seizure. Hum Brain Map. (2017) 38:957–73. 10.1002/hbm.23430 27726245 PMC6866949

[52] Mash LE Linke AC Olson LA Fishman I Liu TT Müller RA. Transient states of network connectivity are atypical in autism: a dynamic functional connectivity study. Hum Brain Map. (2019) 40:2377–89. 10.1002/hbm.24529 30681228 PMC6549695

[53] Sun J Liu Z Rolls ET Chen Q Yao Y Yang W. Verbal creativity correlates with the temporal variability of brain networks during the resting state. Cereb Cortex. (2019) 29:1047–58. 10.1093/cercor/bhy010 29415253

[54] Abrol A Damaraju E Miller RL Stephen JM Claus ED Mayer AR. Replicability of time-varying connectivity patterns in large resting state fMRI samples. Neuroimage. (2017) 163:160–76. 10.1016/j.neuroimage.2017.09.020 28916181 PMC5775892

[55] Laumann TO Snyder AZ Mitra A Gordon EM Gratton C Adeyemo B. On the stability of BOLD fMRI correlations. Cereb Cortex. (2017) 27:4719–32. 10.1093/cercor/bhw265 27591147 PMC6248456

[56] Shen H Li Z Qin J Liu Q Wang L Zeng L-L. Changes in functional connectivity dynamics associated with vigilance network in taxi drivers. Neuroimage. (2016) 124:367–78. 10.1016/j.neuroimage.2015.09.010 26363345

[57] Vidaurre D Smith SM Woolrich MW. Brain network dynamics are hierarchically organized in time. Proc Natl Acad Sci. (2017) 114:12827–32. 10.1073/pnas.1705120114 29087305 PMC5715736

[58] Díez-Cirarda M Strafella AP Kim J Peña J Ojeda N Cabrera-Zubizarreta A. Dynamic functional connectivity in Parkinson's disease patients with mild cognitive impairment and normal cognition. NeuroImage Clin. (2018) 17:847–55. 10.1016/j.nicl.2017.12.013 29527489 PMC5842729

[59] Hutchison RM Womelsdorf T Allen EA Bandettini PA Calhoun VD Corbetta M. Dynamic functional connectivity: promise, issues, and interpretations. Neuroimage. (2013) 80:360–78. 10.1016/j.neuroimage.2013.05.079 23707587 PMC3807588

[60] Rashid B Chen J Rashid I Damaraju E Liu J Miller R. A framework for linking resting-state chronnectome/genome features in schizophrenia: a pilot study. Neuroimage. (2019) 184:843–54. 10.1016/j.neuroimage.2018.10.004 30300752 PMC6230505

[61] Wang J Wang X Xia M Liao X Evans A He Y GRETNA: a graph theoretical network analysis toolbox for imaging connectomics. Front Hum Neurosci. (2015) 9:386 10.3389/fnhum.2015.00458 26175682 PMC4485071

[62] Zalesky A Fornito A Bullmore ET. Network-based statistic: identifying differences in brain networks. Neuroimage. (2010) 53:1197–207. 10.1016/j.neuroimage.2010.06.041 20600983

[63] Stout JC Ellis RJ Jernigan TL Archibald SL Abramson I Wolfson T. Progressive cerebral volume loss in human immunodeficiency virus infection: a longitudinal volumetric magnetic resonance imaging study. Arch Neurol. (1998) 55:161–8. 10.1001/archneur.55.2.161 9482357

[64] Zhou Y Li R Wang X Miao H Wei Y Ali R. Motor-related brain abnormalities in HIV-infected patients: a multimodal MRI study. Neuroradiology. (2017) 59:1133–42. 10.1007/s00234-017-1912-1 28889255

[65] Petras JM. Connections of parietal lobe. J Psychiat Res. (1971) 8:189. 10.1016/0022-3956(71)90018-5 5000110

[66] Melrose RJ Tinaz S Castelo JMB Courtney MG Stern CE. Compromised fronto-striatal functioning in HIV: an fMRI investigation of semantic event sequencing. Behav Brain Res. (2008) 188:337–47. 10.1016/j.bbr.2007.11.021 18242723

[67] Fogassi L Luppino G. Motor functions of the parietal lobe. Curr Opin Neurobiol. (2005) 15:626–31. 10.1016/j.conb.2005.10.015 16271458

[68] Torrey EF. Schizophrenia and the inferior parietal lobule. Schizophr Res. (2007) 97:215–25. 10.1016/j.schres.2007.08.023 17851044

[69] Wagner AD Shannon BJ Kahn I Buckner RL. Parietal lobe contributions to episodic memory retrieval. Trends Cogn Sci. (2005) 9:445–53. 10.1016/j.tics.2005.07.001 16054861

[70] Yamazaki Y Hashimoto T Iriki A. The posterior parietal cortex and non-spatial cognition. F1000 Biol Rep. (2009) 1:74. 10.3410/B1-74 20948614 PMC2948259

[71] Bassett DS Wymbs NF Porter MA Mucha PJ Carlson JM Grafton ST. Dynamic reconfiguration of human brain networks during learning. Proc Natl Acad Sci. (2011) 108:7641–6. 10.1073/pnas.1018985108 21502525 PMC3088578

[72] Barbey AK. Network neuroscience theory of human intelligence. Trends Cogn Sci. (2018) 22:8–20. 10.1016/j.tics.2017.10.001 29167088

[73] Antzoulatos EG Miller EK. Increases in functional connectivity between prefrontal cortex and striatum during category learning. Neuron. (2014) 83:216–25. 10.1016/j.neuron.2014.05.005 24930701 PMC4098789

[74] Bassett DS Yang M Wymbs NF Grafton ST. Learning-induced autonomy of sensorimotor systems. Nat Neurosci. (2015) 18:744–51. 10.1038/nn.3993 25849989 PMC6368853

[75] Duncan J Owen AM. Common regions of the human frontal lobe recruited by diverse cognitive demands. Trends Neurosci. (2000) 23:475–83. 10.1016/S.0166-2236(00)01633-7 11006464

[76] Floyer-Lea A Matthews PM. Distinguishable brain activation networks for short-and long-term motor skill learning. J Neurophysiol. (2005) 94:512–8. 10.1152/jn.00717.2004 15716371

[77] Honda M Deiber M-P Ibánez V Pascual-Leone A Zhuang P Hallett M. Dynamic cortical involvement in implicit and explicit motor sequence learning. A PET study. Brain. (1998) 121:2159–73. 10.1093/brain/121.11.2159 9827775

[78] Braun U Schaefer A Walter H Erk S Romanczuk-Seiferth N Haddad L. Dynamic reconfiguration of frontal brain networks during executive cognition in humans. Proc Natl Acad Sci USA. (2015) 112:11678–83. 10.1073/pnas.1422487112 26324898 PMC4577153

[79] Cramer SC Sur M Dobkin BH O'brien C Sanger TD Trojanowski JQ. Harnessing neuroplasticity for clinical applications. Brain. (2011) 134:1591–609. 10.1093/brain/awr039 21482550 PMC3102236

[80] Mattson MP Moehl K Ghena N Schmaedick M Cheng A Intermittent metabolic switching, neuroplasticity and brain health. Nat Rev Neurosci. (2018) 19:63 10.1038/nrn.2017.156 PMC5913738 29321682

[81] Sarrasin JB Nenciovici L Foisy L-MB Allaire-Duquette G Riopel M Masson S Effects of teaching the concept of neuroplasticity to induce a growth mindset on motivation, achievement, and brain activity: a meta-analysis. Trends Neurosci Educ. (2018) 12:22–31. 10.1016/j.tine.2018.07.003

[82] Tomassini V Matthews PM Thompson AJ Fuglø D Geurts JJ Johansen-Berg H. Neuroplasticity and functional recovery in multiple sclerosis. Nat Rev Neurol. (2012) 8:635–46. 10.1038/nrneurol.2012.179 22986429 PMC3770511

[83] Barbey AK Koenigs M Grafman J. Dorsolateral prefrontal contributions to human working memory. Cortex. (2013) 49:1195–205. 10.1016/j.cortex.2012.05.022 22789779 PMC3495093

[84] Corbetta M Shulman GL. Control of goal-directed and stimulus-driven attention in the brain. Nat Rev Neurosci. (2002) 3:201–15. 10.1038/nrn755 11994752

[85] Levy R Goldman-Rakic PS Segregation of working memory functions within the dorsolateral prefrontal cortex. In: Schneider WX Owen AM Duncan J, editors, Executive Control and the Frontal Lobe: Current Issues. Berlin: Springer (2000). p. 23–32. 10.1007/s002210000397 10933207

[86] Kinoshita M Shinohara H Hori O Ozaki N Ueda F Nakada M. Association fibers connecting the Broca center and the lateral superior frontal gyrus: a microsurgical and tractographic anatomy. J Neurosurg. (2012) 116:323–30. 10.3171/2011.10.JNS11434 22077454

[87] Li W Qin W Liu H Fan L Wang J Jiang T. Subregions of the human superior frontal gyrus and their connections. Neuroimage. (2013) 78:46–58. 10.1016/j.neuroimage.2013.04.011 23587692

[88] Leech R Kamourieh S Beckmann CF Sharp DJ. Fractionating the default mode network: distinct contributions of the ventral and dorsal posterior cingulate cortex to cognitive control. J Neurosci. (2011) 31:3217–24. 10.1523/JNEUROSCI.5626-10.2011 21368033 PMC6623935

[89] Chang L Speck O Miller EN Braun J Jovicich J Koch C. Neural correlates of attention and working memory deficits in HIV patients. Neurology. (2001) 57:1001–7. 10.1212/WNL.57.6.1001 11571324

[90] Keltner JR Connolly CG Vaida F Jenkinson M Fennema-Notestine C Archibald S. HIV distal neuropathic pain is associated with smaller ventral posterior cingulate cortex. Pain Med. (2017) 18:428–40. 10.1093/pm/pnw180 27497320 PMC6074843

[91] Wang X Foryt P Ochs R Chung J-H Wu Y Parrish T. Abnormalities in resting-state functional connectivity in early human immunodeficiency virus infection. Brain Connect. (2011) 1:207–17. 10.1089/brain.2011.0016 22433049 PMC3621309

[92] Wilson TW Heinrichs-Graham E Becker KM Aloi J Robertson KR Sandkovsky U. Multimodal neuroimaging evidence of alterations in cortical structure and function in HIV-infected older adults. Hum Brain Map. (2015) 36:897–910. 10.1002/hbm.22674 25376125 PMC4491915

[93] McBride JA Striker R. Imbalance in the game of T cells: what can the CD4/CD8 T-cell ratio tell us about HIV and health? PLoS Pathog. (2017) 13:e1006624. 10.1371/journal.ppat.1006624 29095912 PMC5667733

[94] Sauter R Huang R Ledergerber B Battegay M Bernasconi E Cavassini M. CD4/CD8 ratio and CD8 counts predict CD4 response in HIV-1-infected drug naive and in patients on cART. Medicine. (2016) 95:e5094. 10.1097/MD.0000000000005094 27759638 PMC5079322

[95] Zhuang Y Qiu X Wang L Ma Q Mapstone M Luque A. Combination antiretroviral therapy improves cognitive performance and functional connectivity in treatment-naive HIV-infected individuals. J Neurovirol. (2017) 23:704–12. 10.1007/s13365-017-0553-9 28791662 PMC5655604

[96] Sanfratello L Houck JM Calhoun VD. Relationship between MEG global dynamic functional network connectivity measures and symptoms in schizophrenia. Schizophr Res. (2019) 209:129–34. 10.1016/j.schres.2019.05.007 31130399 PMC6661190

[97] Büttner A Mehraein P Weis S. Vascular changes in the cerebral cortex in HIV-1 infection. Acta Neuropathol. (1996) 92:35–41. 10.1007/s004010050486 8811123

[98] Cardenas V Meyerhoff D Studholme C Kornak J Rothlind J Lampiris H Evidence for ongoing brain injury in human immunodeficiency virus-positive patients treated with antiretroviral therapy. J Neurovirol. (2009) 15:324–33. 10.1080/13550280902973960 19499454 PMC2889153

[99] Everall IP Luthert PJ Lantos PL. Neuronal number and volume alterations in the neocortex of HIV infected individuals. J Neurol Neurosurg Psychiatry. (1993) 56:481–486. 10.1136/jnnp.56.5.481 8505639 PMC1015005

[100] Li R Wang W Wang Y Peters S Zhang X Li H. Effects of early HIV infection and combination antiretroviral therapy on intrinsic brain activity: a cross-sectional resting-state fMRI study. Neuropsychiatr Dis Treat. (2019) 15:883. 10.2147/NDT.S195562 31114203 PMC6497505

[101] Yu X Gao L Wang H Yin Z Fang J Chen J. Neuroanatomical changes underlying vertical HIV infection in adolescents. Front Immunol. (2019) 10:814. 10.3389/fimmu.2019.00814 31110499 PMC6499204

[102] Küper M Rabe K Esser S Gizewski E Husstedt I Maschke M. Structural gray and white matter changes in patients with HIV. J Neurol. (2011) 258:1066–75. 10.1007/s00415-010-5883-y 21207051

[103] Crowell CS Malee KM Yogev R Muller WJ. Neurologic disease in HIV-infected children and the impact of combination antiretroviral therapy. Rev Med Virol. (2014) 24:316–31. 10.1002/rmv.1793 24806816

[104] Saylor D Dickens AM Sacktor N Haughey N Slusher B Pletnikov M HIV-associated neurocognitive disorder—pathogenesis and prospects for treatment. Nat Rev Neurol. (2016) 12:234 10.1038/nrneurol.2016.27 26965674 PMC4937456

[105] Toich JTF Taylor PA Holmes MJ Gohel S Cotton MF Dobbels E. Functional connectivity alterations between networks and associations with infant immune health within networks in HIV infected children on early treatment: a study at 7 years. Front Hum Neurosci. (2017) 11:635. 10.3389/fnhum.2017.00635 29375341 PMC5768628

[106] Becker JT Maruca V Kingsley LA Sanders JM Alger JR Barker PB. Factors affecting brain structure in men with HIV disease in the post-HAART era. Neuroradiology. (2012) 54:113–21. 10.1007/s00234-011-0854-2 21424708 PMC3154580

