# Supplementary material for: Atypical Resting-State Functional Connectivity Dynamics Correlate With Early Cognitive Dysfunction in HIV Infection
Source: Front Neurol. 2021 Jan 14;11:606592. doi: 10.3389/fneur.2020.606592 (PMC7841016; doi:10.3389/fneur.2020.606592)
Supplement: Supplementary file 1 [file Data_Sheet_1.docx]

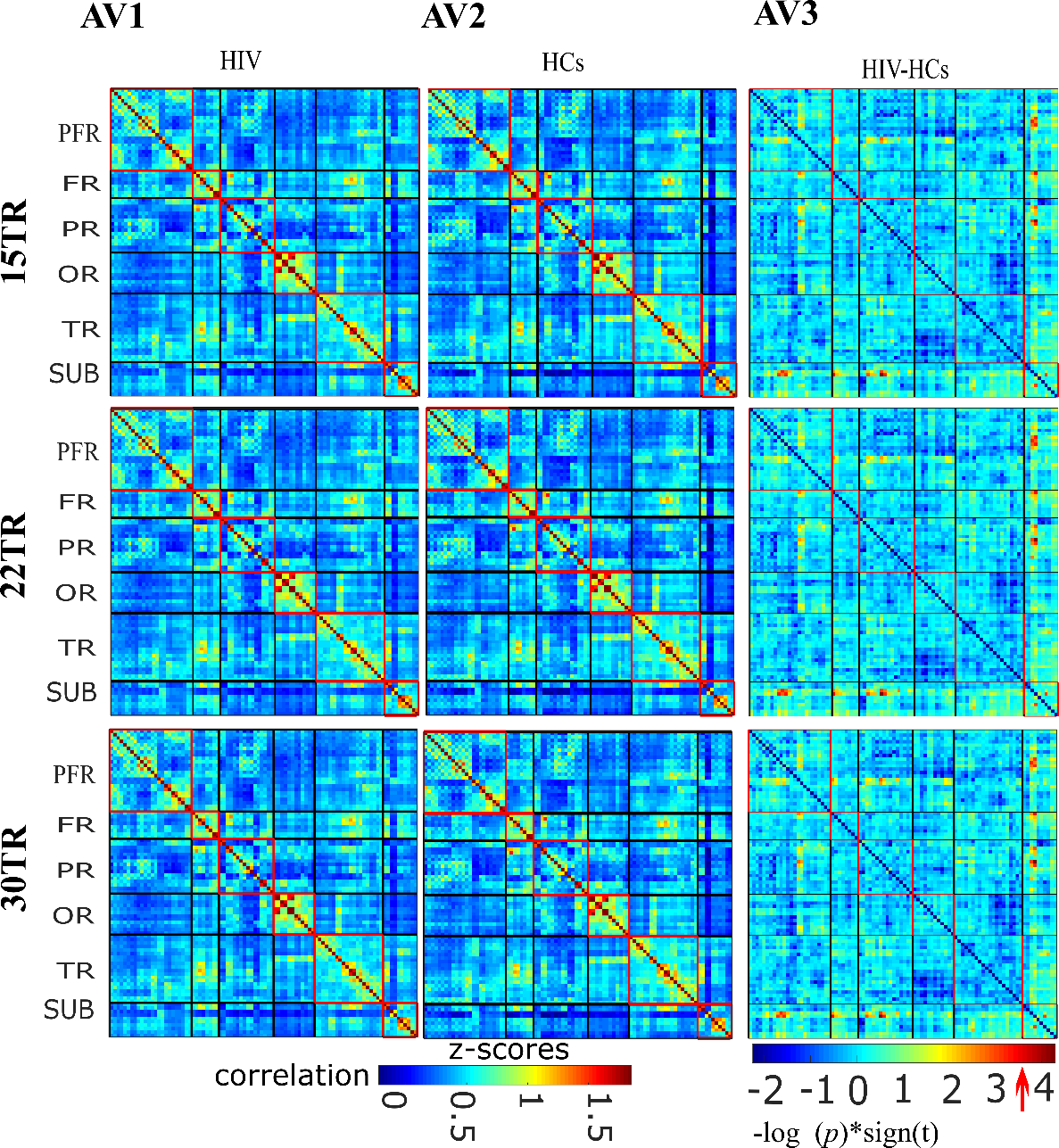


**Supplementary Figure 1.** The illustration of reproducibility of average dynamic RSFC maps at different window sizes (15TR, 22TTR, and 30TR)**.** (**AV1-AV2**) Mean dynamic RSFC maps for healthy controls (column, **AV1**) and patients with HIV (column, **AV2**). Thick black lines partition the RSFC maps into six subcategories (i.e., prefrontal (PFR), other frontal (FR), parietal (PR), occipital (OR), temporal and subcortical regions. (**AV3**) The group difference (HIV–HCs) of dynamic RSFC (AV1-AV2). Values are plotted as−log10 (p-value) ×sign (t-statistic). Statistical significance was achieved at *P*<0.05.
