# Supplementary material for: Atypical Resting-State Functional Connectivity Dynamics Correlate With Early Cognitive Dysfunction in HIV Infection
Source: Front Neurol. 2021 Jan 14;11:606592. doi: 10.3389/fneur.2020.606592 (PMC7841016; doi:10.3389/fneur.2020.606592)
Supplement: Supplementary file 2 [file Data_Sheet_2.docx]

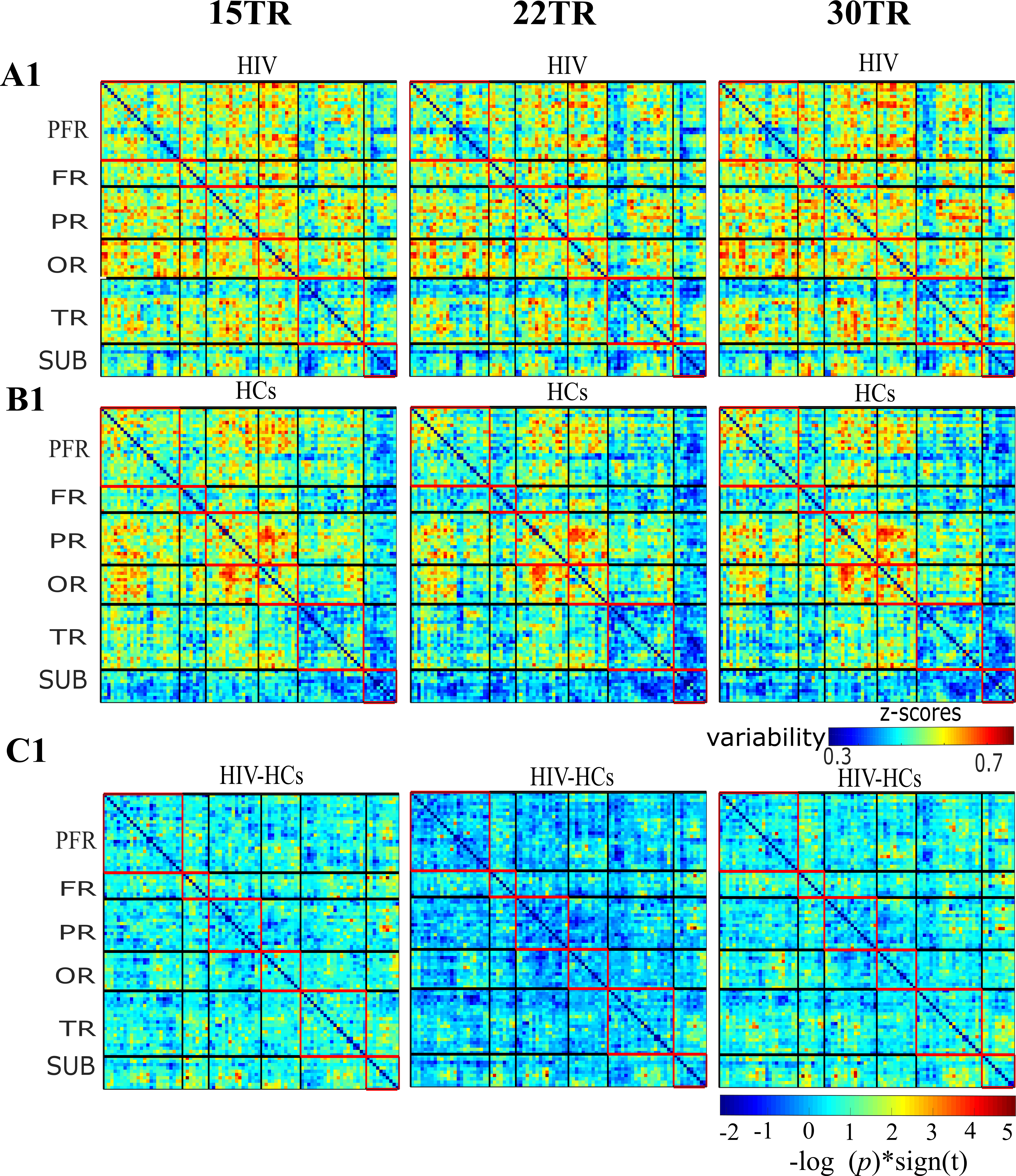


**Supplementary Figure 2**. The illustration of reproducibility of temporal variabilities in different windows size (15TR, 22TR, 30TR). (**A1**) The mean variability maps for HIV patients (HIV) at 5TR, 22TR, and 30TR. (**B1**) the mean variability for healthy controls (HCs). (**C1**) The group differences (HIV–HCs) in connectivity variabilities.
