# Supplementary material for: Atypical Resting-State Functional Connectivity Dynamics Correlate With Early Cognitive Dysfunction in HIV Infection
Source: Front Neurol. 2021 Jan 14;11:606592. doi: 10.3389/fneur.2020.606592 (PMC7841016; doi:10.3389/fneur.2020.606592)
Supplement: Supplementary file 3 [file Data_Sheet_3.docx]

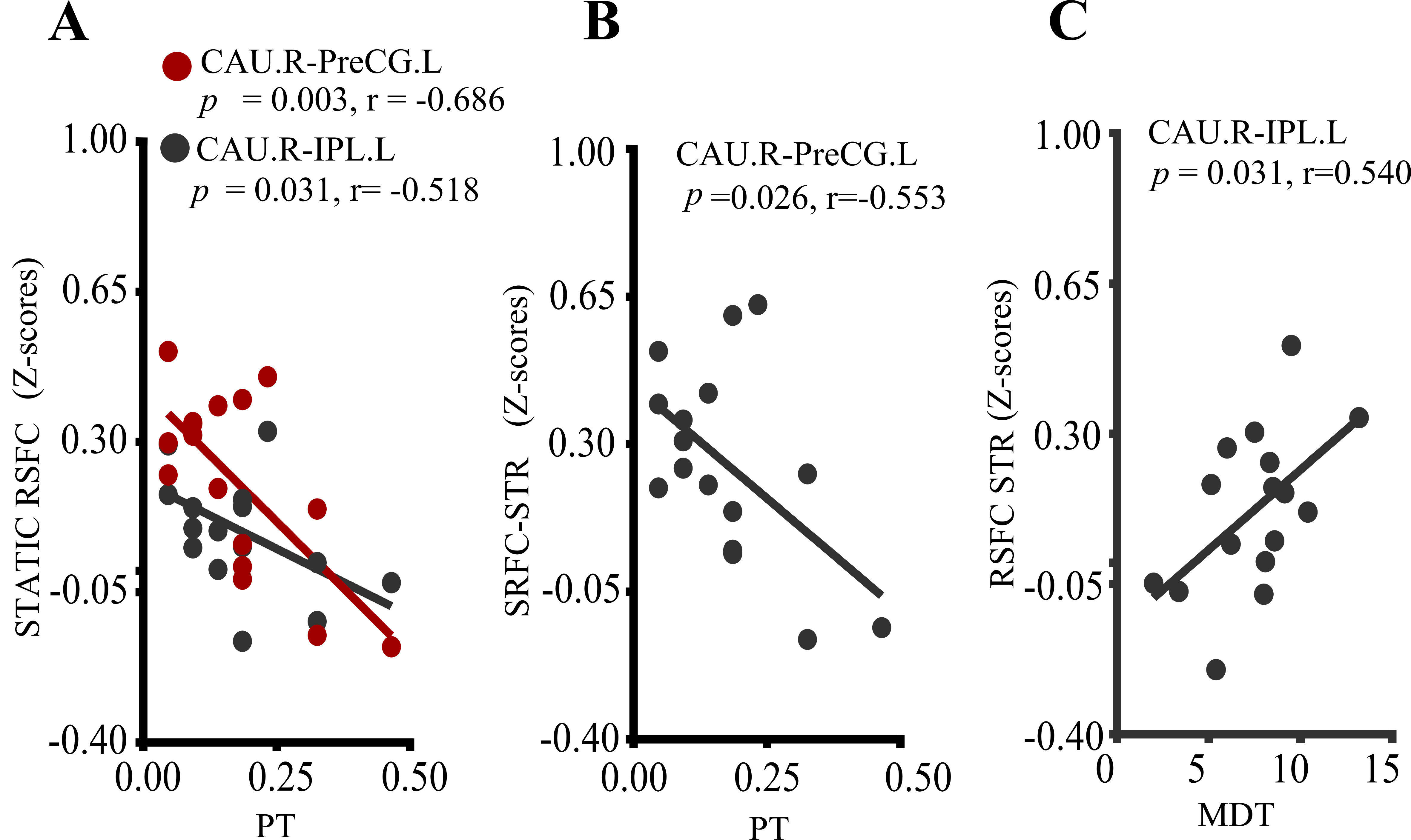


**Supplementary Figure 3.** Correlations of state properties with static and dynamic RSFC in HIV. (**A**) Reduced state-2 transitioning (PT) was negatively associated with the static RSFC of CAU.R-PreCG.L (red) and CAU.R-IPL.L (black). (**B**) The average dynamic RSFC of CAU.R-PreCG.L was also related to reduced transitioning (PT) of state 4. (**C**) The positive relationship between the mean dwelling time of the state with weaker occipital connectivity (state 2) and average dynamic CAU.R-IPL.L RSFC. *P*, *P*-value; r, Pearson’s correlation coefficient; R, right; L, left; CAU, the caudate nucleus; PreCG, precentral gyrus; IPL, inferior parietal lobe. Note: correlation was significant at p<0.05.
