# Supplementary material for: Atypical Resting-State Functional Connectivity Dynamics Correlate With Early Cognitive Dysfunction in HIV Infection
Source: Front Neurol. 2021 Jan 14;11:606592. doi: 10.3389/fneur.2020.606592 (PMC7841016; doi:10.3389/fneur.2020.606592)
Supplement: Supplementary file 4 [file Table_1.docx]

**Supplementary Table 1**. MRI parameter settings

| Imaging modality | Parameter configurations |
| --- | --- |
| T1-weighted anatomical images | Magnetic field strength=3T;  3D-inversion recovery;  repetition time(TR)=1900ms;  echo time(TE)=2.52ms; |
|  | inversion time = 900 ms;  flip angle = 9°;  field of view=250 × 250 mm2;  Matrix size = 246×256;  slice thickness = 1 mm;  and voxel size =1 ×0.977 ×0.977 mm3. |
| Resting-state functional fMRI images | Repetition time/echo time ratio = 2000/30ms; |
|  | flip angle = 90°;  field of view =224 × 224 mm2;  matrix size =64 × 64;  voxel size =4.2 × 4.2 × 4.2mm3, and |
|  | Slice thickness = 3.5 mm. |
