# Supplementary material for: Atypical Resting-State Functional Connectivity Dynamics Correlate With Early Cognitive Dysfunction in HIV Infection
Source: Front Neurol. 2021 Jan 14;11:606592. doi: 10.3389/fneur.2020.606592 (PMC7841016; doi:10.3389/fneur.2020.606592)
Supplement: Supplementary file 5 [file Table_2.docx]

**Supplementary Table 2**. Methods for assessment of six cognitive domains.

| Domain | Evaluation Test(Testing method) |
| --- | --- |
| Learning and Recall (Memory) | The Hopkins Verbal Learning Test–Revised (HVLT-R), and The Brief Visuospatial Memory Test–Revised (BVMT-R) |
| Motor function | The Grooved Pegboard test |
| Abstract/executive function | The Wisconsin Card Sorting Test-64 (WCST-64) |
| verbal/ language | Category fluency and animal naming test |
| Attention/ working memory | The Continuous Performance Test Identical Pairs (CPT-IP), The Wechsler Memory Scale-III (WMS-III), and Paced Auditory Serial Addition Test (PASAT) |
| Information processing speed | The trail making test part A |
