# Supplementary material for: Atypical Resting-State Functional Connectivity Dynamics Correlate With Early Cognitive Dysfunction in HIV Infection
Source: Front Neurol. 2021 Jan 14;11:606592. doi: 10.3389/fneur.2020.606592 (PMC7841016; doi:10.3389/fneur.2020.606592)
Supplement: Supplementary file 6 [file Table_3.docx]

**Supplementary Table 3**. Metrics for dynamic RSFC and state properties

| Metrics | Mathematical representations |
| --- | --- |
| Dynamic RSFC properties |  |
| Average dynamic RSFC across temporal windows(RSFC-STR) | $RSFC-\mathrm{STR}_{n} ={RSFC}_{m}=\frac{1}{N}\sum_{i}^{N} {RSFC}_{w_{i}}$ |
| Temporal variability(RSFC-SD) | $RSFC-\mathrm{SD}_{n} =\sqrt{\frac{1}{N-1}\sum_{i=1}^{N} {({RSFC}_{w_{i}}-{RSFC}_{m})}^{2}}$ |
| State properties |  |
| Mean Dwelling Time(MDT) | $\mathrm{MD}T_{s\mathrm{tate}_{k}}=\frac{1}{T}(\sum_{t=1}^{T} \left( {Endstate}_{k}-Start{state}_{k}+1 \right))$ |
| Probability of transition (PT) | $PT_{s\mathrm{tate}_{k}}=\frac{\sum_{j=1}^{K} {transition}_{{state}_{k}},k\neq j}{\sum transitions}$ |
| Probability of state-to-state transitioning(PSPT) | $\mathrm{PSP}T_{s\mathrm{tate}_{j\to k}}=\frac{\sum{transitionsstate}_{j\to k}}{\sum transitions}$ |
|  |  |

Note: *n*, is for every subject *n;* RSFC*_m,_* average dynamic RSFC; N, number of windows; *w_i_*, window *i*; state*_k_*_,_ state number *k* ; MDT_statek_, mean dwelling time of the state k for every time t a RSFC map enters a state k; PT_Statek_, probability of transitioning to state *k*; PSPT*_statej→k_* , the probability of transitioning from state *j* to state *k*.
